# Revealing the endocrine landscape of INSL3/RXFP2 signaling in hamster

**DOI:** 10.1371/journal.pone.0329696

**Published:** 2025-08-12

**Authors:** Aidet Ruiz, Luis Ramos

**Affiliations:** Department of Reproductive Biology, Instituto Nacional de Ciencias Médicas y Nutrición Salvador Zubirán, México City, México; Universite Clermont Auvergne, FRANCE

## Abstract

In mammals, insulin-like peptide 3 (INSL3) and its cognate receptor (RXFP2) are reported to be essential regulators of male reproductive physiology. It is also believed that INSL3/RXFP2 signaling has a role in female ovarian function and follicle development, although its exact mechanisms and functions are still being studied. This research aimed to explore the transcriptional landscape of *INSL3*/*RXFP2* genes in adult hamsters. The cloned cDNA fragments of INSL3/RXFP2 were 888 and 3233 base-pairs (bp), including an open reading frame of 375 bp and 2211 bp, encoding 125 and 737 amino acids, respectively. The phylogenetic relationships, gene function predictions, and three-dimensional structure predictions of INSL3/RXFP2 revealed evolutionary conserved domains. Quantification analysis showed that INSL3 mRNA was highly expressed in the testis, male adrenal glands, and ovary, weakly expressed in male reproductive glands and the female adrenal gland, and barely expressed in the male hypothalamus. INSL3 mRNA expression peaked in ovaries during the proestrus phase, indicating sex-steroid-dependent regulation. RXFP2 mRNA was mainly expressed in the male hypothalamus and uterus, slightly lower in the female cerebellum, epididymis, and ovary, and much lower in the female hypothalamus, male cerebellum, and testis. The RXFP2 in the uterus exhibited the highest expression during the proestrus phase, suggesting regulation via sex steroids. Our findings suggest that sexually dimorphic INSL3/RXFP2 signaling plays a novel role in the reproductive and endocrine systems, particularly in uterine tissue. These results suggest that INSL3/RXFP2 signaling in the hamster model may provide an alternative avenue for studying human female reproduction and endocrine disorders in conditions such as endometriosis or uterine disease in future clinical research.

## Introduction

The Insulin-like 3 peptide hormone (INSL3) and its cognate relaxin-family peptide receptor 2 (RXFP2) are predominantly synthesized and expressed in testicular Leydig cells. In mammalian tissues, the INSL3/RXFP2 signaling pathway plays a decisive role in male reproductive physiology [1,2]. During mouse embryogenesis, this pathway has a physiological function in the development of the gubernaculum ligament for the initial transabdominal descent of the testis, a condition common in placental mammals [3–5]. Adult Insl3 − / − mice have shown impaired testicular descent due to gubernaculum developmental abnormalities, which results in abnormal spermatogenesis and infertility [6,7]. Moreover, INSL3 has been reported as a biomarker of Leydig cell functional capacity in adult male mice [8]. Concurrently, genomic analysis has revealed that human gene mutations in *INSL3* and *RXFP2* cause testicular torsion and cryptorchid testes, respectively [9,10]; however, genetic analysis in a Greek pediatric cohort suggests that *INSL3* gene mutations are not a common cause of testicular maldescent in humans [11]. Recently, mutation studies in human patients with bilateral cryptorchidism and male infertility reported bi-allelic loss-of-function (LoF) variants in INSL3 and RXFP2, while carriers of the heterozygous variant remain phenotypically unaffected [12]. Collectively, these studies have indicated a potential role of *INSL3* and *RXFP2* genes in male pathophysiology; therefore, future assays are crucial to establish a direct cause-effect relationship.

However, in female mammals, it has been shown that INSL3 is synthesized in the steroidogenic theca interna cells of antral follicles [13]. The *INSL3* gene is also expressed in the human corpus luteum, trophoblast [14], mammary glands [15], and endometrium [16]; while the *RXFP2* gene expression has been identified in the fetal membranes and placenta [17]. In bovines, RXFP2 mRNA expression has been observed in theca cells, luteal cells, and oocytes [18,19]. Therefore, a biological role has been suggested for the INSL3/RXFP2 signaling pathway in maintaining healthy uterine function. RXFP2 is also intimately involved in female reproductive processes. It is expressed in the ovaries and has been implicated in follicle development, ovulation, and the regulation of the menstrual cycle [13].

Overall, the INSL3/RXFP2 signaling pathway is associated with reproductive health and may have implications for fertility and reproductive disorders. Ongoing molecular studies on this pathway continue to shed light on their physiological functions. However, further studies are required to fully comprehend its potential therapeutic value in the treatment of various diseases and possible clinical applications. The exact gene expression and potential physiological role of the INSL3/RXFP2 signaling pathway in females remain unclear, mandating more experimental assays to completely understand the signaling mechanisms of INSL3/RXFP2 in various tissues and their biological relevance. Additionally, DNA cloning and differing expression levels of INSL3 between various biological species remain largely unexplored. Thus, the current study aims to ascertain the gene expression profiles of the INSL3/RXFP2 signaling pathway cloned from a rodent model, such as hamsters (*Mesocricetus auratus*). Preceding reports in this biological species, specifically in the Harderian gland (HG), described a sexual dimorphism controlled by the levels of sex-steroid hormones, making it a compelling model to study the molecular mechanism of gene regulation by sex-steroid hormones and their physiological effects. [20]. There are some relevant reasons for selecting the hamster as a model species for investigating INSL3 and RXFP2 expression. Hamsters have well-defined seasonal reproductive cycles, which allows for the study of how the expressions of genes such as *INSL3* and *RXFP2* vary at different stages of gonadal development, as well as reproductive tissues regulated by steroid/protein hormones. Therefore, studying their regulation in hamsters may provide information that could be extrapolated to other species, including humans [21,22].

## Materials and methods

### Hamster tissues, RNA analysis, and cDNA synthesis

All experimental assays involving hamsters (*Mesocricetus auratus*) were approved by our institution’s ethical committee (INCMNSZ, BRE-1930-18-19-1). The hamsters used in this study were offspring of a colony maintained at Universidad Autónoma Metropolitana-Xochimilco (UAM-X; México City, México; code number AUT-B-C-0215–016). We used adult hamsters (10 months old; 150–200 g; 6 inches in length), with five males, and five females each in proestrus, estrus, metestrus, and diestrus, who all had access to food and water ad libitum. The environment was regulated for temperature, humidity, and a 12-h light/darkness cycle. The stage of the estrous cycle was confirmed via standard vaginal smear examination. The hamsters were anesthetized with ketamine:xylazine (80 mg/kg:8 mg/kg, intramuscularly). Male and female adult hamsters were sacrificed by decapitation; we procured samples from the testis, ovary, uterus, lung, heart, pancreas, adrenal glands, liver, epididymis, duodenum, spleen, brain, and HG for tissue distribution detection. These tissues were immediately placed on dry ice and then stored in a −70 °C freezer.

Total RNA was isolated using TRIzol Reagent (Invitrogen, Carlsbad, CA, USA) according to the manufacturer’s protocol. It was collected in diethyl pyrocarbonate (DEPC)-treated distilled water. RNase-free DNase I was added to the total RNAs to remove genomic DNA. The quantity and quality of total RNA were evaluated by spectrophotometry at an A_260_/A_280_ ratio of 1.8 (Beckman DU 650, Fullerton, CA, USA) through duplicate samples for each tissue. Additionally, its integrity (20 µg) was assessed based on the localization of ribosomal RNA (28S:18S rRNA ratio) using denaturing formaldehyde/MOPS/1.5% agarose electrophoresis. The quantity, quality, and integrity of the isolated total RNA were deemed sufficient for subsequent cDNA synthesis. Following the manufacturer’s recommendation, 2 µg of the total RNA was used to synthesize single-stranded cDNA using both a Transcriptor First Strand cDNA Synthesis kit for cloning (Roche Diagnostics, IN, USA) and a Maxima First Strand cDNA Synthesis kit (ThermoScientific, Vilnius, Lithuania) for real-time quantitative PCR (qPCR). The synthesized first-strand cDNAs were stored at −20 °C until further analysis was performed.

### 5′ and 3′ Rapid Amplification of cDNA Ends (RACE)

Full-length cDNA sequences of the *INSL3* and *RXFP2* genes were isolated from hamster testis by RACE, following the instructions for the SMARTer® RACE 5′/3′ kit (Takara Bio Inc, Mountain View, CA, USA). The 5´ and 3´ end cDNA sequences of INSL3 and RXFP2 were obtained via RACE, using 5 µg of total RNA extracted from hamster testis. Four gene-specific primers (GSPs) based on known sequences (*Mus musculus*, *Rattus norvegicus*, and *Homo sapiens*) of conserved regions were designed for RACE (INSL3: RACE 5′ = 5´-ccacagagcttgtcacgcgcctcag-3´, RACE 3′ = 5′-ctcaccggctgcacccagcaagac-3´; RXFP2: RACE 5′ = 5´-gtcaggacgatgacatgaagtag-3´, RACE 3′ = 5´-gggacaatataatgaagccagtgtc-3´). PCR bands were separated by 1.2% agarose gel electrophoresis, and the gel was cut under a transilluminator. The PCR-amplified DNA fragments were recovered according to the instructions of the GeneJET Gel Extraction Kit (Thermo Fisher Scientific, Vilnius, Lithuania). The PCR products obtained by RACE were sequenced, and four specific primers (INSL3: 5´-cgaccttgtgggtgctg-3´, 5´-gggcatgtgaccatccttt-3´; RXFP2: 5´-gggaggcaccagactcta-3´, 5´-gtctcagacgccatcttcc-3´) were designed to amplify the full-length cDNA of INSL3 and RXFP2.

### Full-length cDNA cloning

The PCR products isolated through RACE were cloned using a TOPO TA cloning kit for sequencing, following the manufacturer’s instructions (Invitrogen Co., Carlsbad, CA, USA). After transforming into competent *Escherichia coli* DH5α cells, the pCR2.1-TOPO TA Vector-Taq-amplified PCR products were purified as per the instructions given by the E.Z.N.A. Plasmid DNA Mini and Maxi kits (Omega Bio-Tek, Inc., Norcross, Georgia, USA). The positive clones were selected for Sanger sequencing.

### DNA sequencing and bioinformatic technologies

Full-length cDNA sequences were sequenced bidirectionally (in both sense and antisense directions) using a BigDye Terminator v3.1 Cycle Sequencing kit (Applied Biosystems, Austin, TX, USA). This was carried out with an initial denaturation at 96 °C for 1 min, followed by 35 cycles at 96 °C for 10 s, 50 °C for 5 s, and 60 °C for 4 min (Veriti 96 well Thermal Cycler, Applied Biosystems, Marsiling, Singapore). Reactions were purified using a BigDye XTerminator™ Purification kit (Applied Biosystems, Austin, TX, USA) according to the manufacturer’s instructions. The products from the sequencing reaction were electrophoresed using an ABI-PRISM 310 genetic analyzer (Applied Biosystems, Foster City, CA, USA). The conditions for sequencing included a temperature of 50 °C, injection voltage of 15 kV, injection time of 5–7 s, and a current of 5–8 μA. All the obtained nucleotide sequence data was analyzed using Chromas sequencing software version 2.6.6. The INSL3/RXFP2 amino acid sequences were deduced via the Expert Protein Analysis System. ExPaSy proteomic tools were utilized to predict the isoelectric point and molecular weight of INSL3/RXFP2. The sequence data were compared using the Basic Local Alignment Search Tool and the Clustal Omega program. The DeepLoc-2.1 was used for the prediction of eukaryotic protein subcellular localization. SignalP-2.0 was used to predict signal peptides and their cleavage site. TMHMM – 2.0 was used to identify the prediction of transmembrane helices. The evolutionary history was inferred using the Maximum Likelihood method, the JTT matrix-based model, and the Molecular Evolutionary Genetics Analysis (MEGA) software. Three-dimensional (3D) models of INSL3/RXFP2 were generated with the Robetta software package. The homology model was rendered using PyMOL version 2.3 to observe the 3D structure of INSL3/RXFP2.

### qPCR assays

Gene expression levels of hamster INSL3/RXFP2 were assessed via qPCR from various tissues. The assays were performed using 2 μg total RNA and the Maxima First Strand cDNA Synthesis kit (ThermoScientific, Vilnius, Lithuania), as per the instructions. Briefly, every qPCR reaction (20 μL) included 0.2 μL of 10 µM probe library, 0.2 μL of 20 µM forward primer, 0.2 μL of 20 µM reverse primer (INSL3 = 5′-tgacaagctctgtggccac-3′ and 5′-caagtgcatgcaggagctg-3′; RXFP2 = 5′-acttccagtcaaagttttcagcaaa-3′ and 5′-aaaaatgccttcctggatatgtgtg-3′), 4.0 μL of TaqMan Master LightCycler, and 5.0 μL of cDNA template. *INSL3*/*RXFP2* gene expressions were detected on a LightCycler 2.0 instrument (Roche Diagnostics, Indianapolis, IN, USA). The *β-*actin** (*ACTB*) gene (5′-agctatgagctgcctgatgg-3′ and 5′-caggaaggaaggctggaaa-3′) was used as the internal reference. Amplification was done with 45 cycles of 95 °C for 10 s and 60 °C for 30 s, and 72 °C for 1 second. Each sample was set up for five independent biological replicates, and the relative expression levels were determined using the 2^−ΔΔCT^ method.

### Statistical data analysis

Data are presented as the mean and standard deviation (SD) of five animals per group. GraphPad Prism software (California Corporation, USA) was used to analyze and graph gene expressions. One-way analysis of variance (ANOVA) was used to compare groups under different physiological conditions, taking into account both sex and group. A *P*-value < 0.05 was considered statistically significant. Additionally, an ANOVA with a *post hoc* Tukey test was conducted to compare means between different tissues in male and female hamsters. Data were also evaluated with *post hoc* Sidak tests during the estrous cycle.

## Results

### Molecular structure of INSL3 and RXFP2

**Fig 1:**
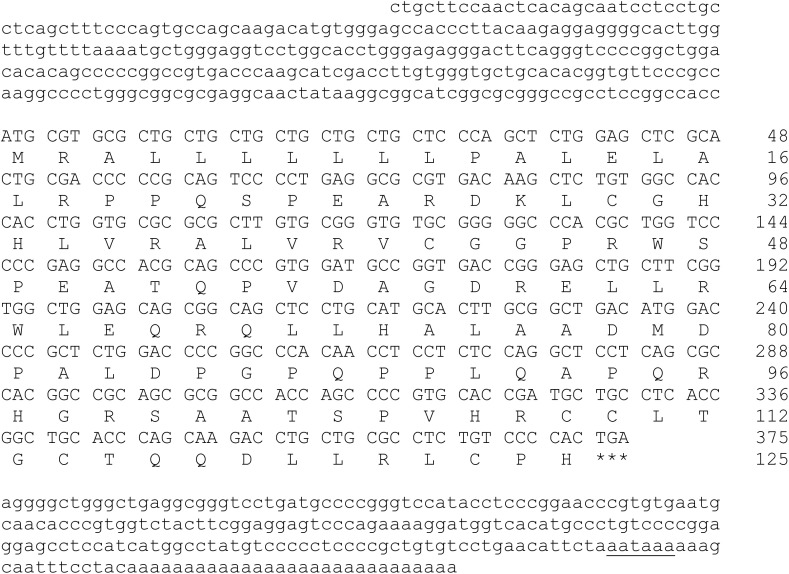
Identification of INSL3 full-length cDNA and deduced amino acid sequence in hamster, *Mesocricetus auratus.* The initiation codon of ATG and stop codon TGA are indicated in bold. The polyadenylation signal AATAAA is underlined. Numbers indicate the amino acid and nucleotide positions.

**Fig 2:**
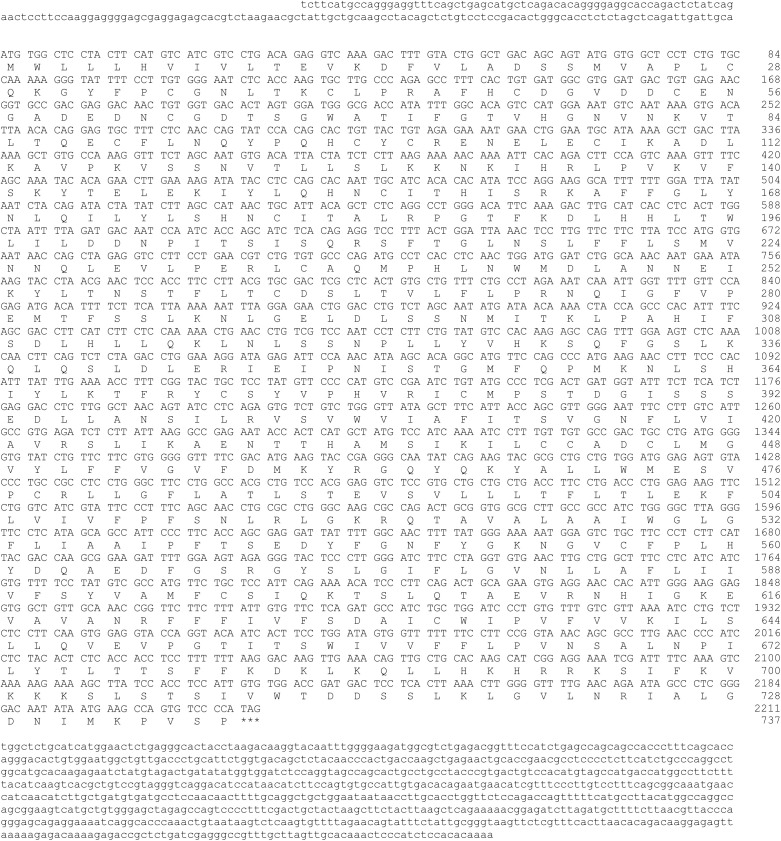
Sequence analyses of the cDNA encoding *Mesocricetus auratus* RXFP2. The nucleotides and amino acids are numbered from right to left. The positions of the cDNA and deduced amino acid sequences are indicated by numbers on the right. The start codon was ATG and the stop codon is marked by asterisks (***).

In this study, Sanger sequence analysis revealed that the cloned sequences of INSL3 and RXFP2 from hamsters are 888 bp and 3233 bp in length, respectively. A 281 bp 5′-untranslated region (UTR) and a 229 bp 3′-UTR with a putative polyadenylation consensus signal (AATAAA) were identified in INSL3, while a 175 bp 5′-UTR and an 844 bp 3′-UTR were identified in RXFP2. Additionally, the cDNAs of INSL3 (GenBank accession number PQ606061) and RXFP2 (GenBank accession number PQ606062) contain open reading frames (ORFs) of 375 bp (Fig 1) and 2211 bp (Fig 2) encoding 125 amino acids (molecular weight 13786) with a theoretical pI value of 8.33 and 737 amino acids (molecular weight 83270) with a theoretical pI value of 8.71, respectively.

**Fig 3:**
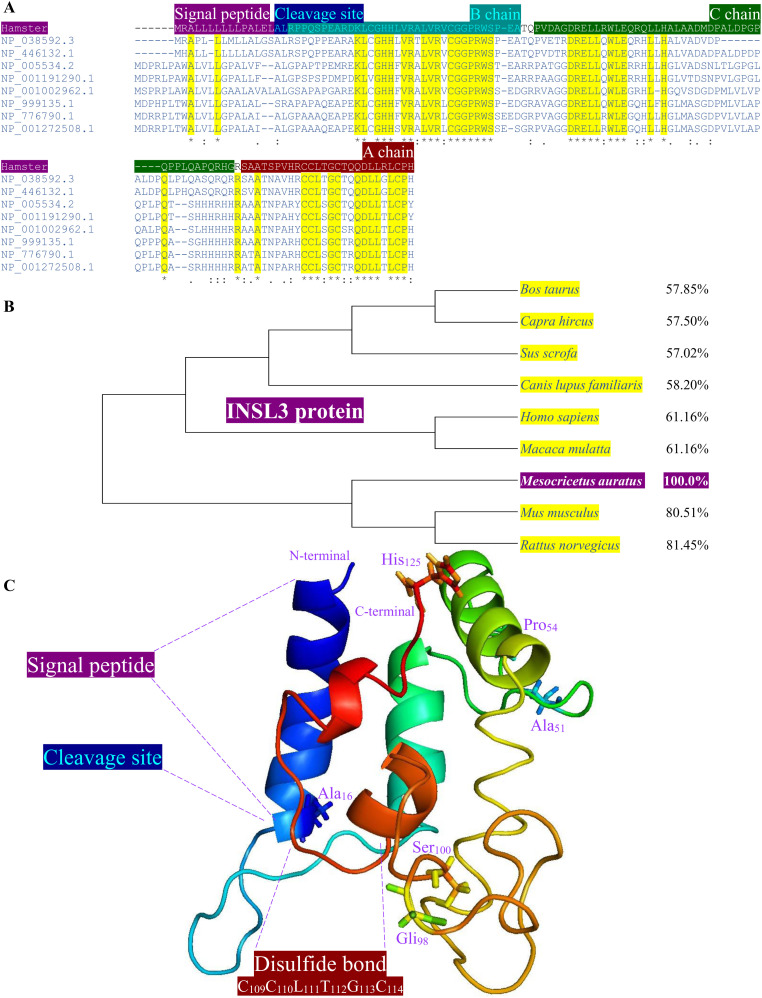
Multiple alignment of the INSL3 amino acid sequence from hamster and other mammals. **(A)** The alignment of INSL3 amino acid sequence from *B. taurus* (NP_776790.1), *C. hircus* (NP_001272508.1), *S. scrofa* (NP_999135.1), *C. lupus familiaris* (NP_001002962.1), *H. sapiens* (NP_005534.2), *M. mulatta* (NP_001191290.1), *M. musculus* (NP_038592.3), and *R. norvegicus* (NP_446132.1). Asterisks and boxed yellow indicate conserved residues. Structural domains are indicated by boxed colors. **(B)** Evolutionary analysis of INSL3 from hamster using the Maximum Likelihood method and JTT matrix-based model in MEGA **X.** This analysis involved 9 amino acid sequences with percentage similarities ranging from 57.02% to 81.45%. The hamster INSL3 was clustered with the Rodentia group. **(C)** Detailed structural analyses, including INSL3 3D structure obtained computational modeling, indicated key insights into the peptide signal; B-chain (Ala_16_–Glu_51_), C-chain (Pro_54_–Gli_98_), and A-chain (Ser_100_–His_125_); disulfide bond region; and receptor binding mechanisms (B-chain).

**Fig 4:**
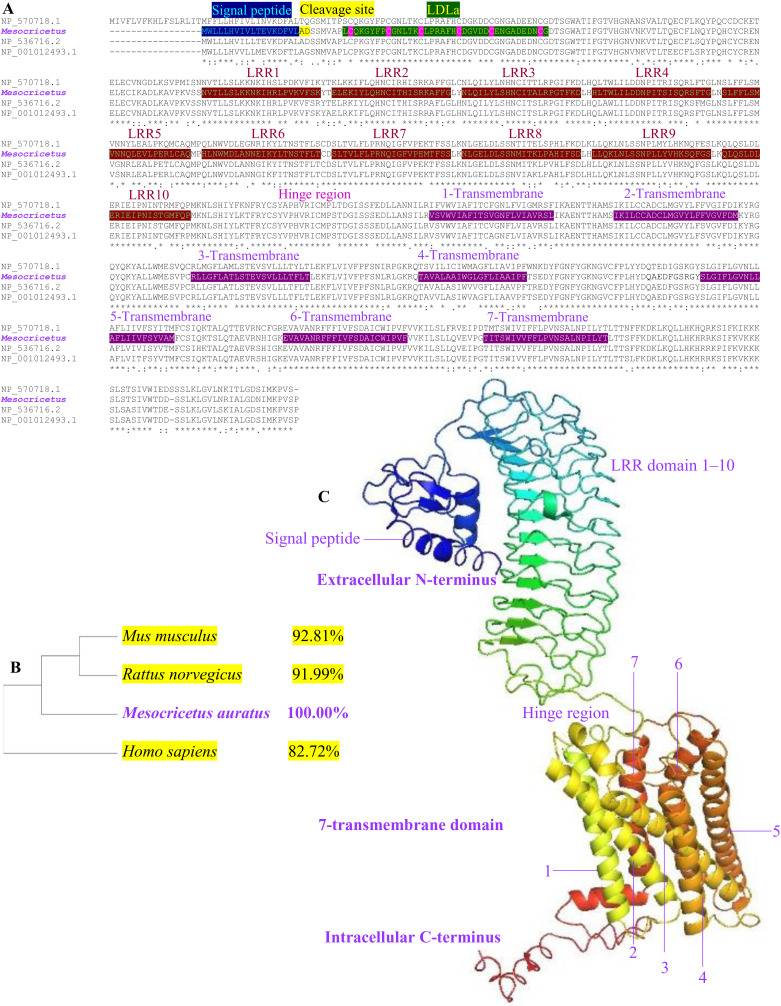
Molecular and bioinformatic analysis of RXFP2 in hamster, *Mesocricetus auratus.* **(A)** Multiple amino acid sequence alignment of RXFP2 of hamster with other representative mammals, *H. sapiens* (NP_570718.1), *R. norvegicus* (NP_001012493.1), and *M. musculus* (NP_536716.2). Identical and similar amino acids are marked with asterisks. The deduced amino acids were predicted to contain conserved domains of the RXFP2, such as a signal peptide (1–19 residues), cleavage site (19–20 residues), low-density lipoprotein receptor class A domain (LDLa, 27–64 residues) which contains six disulphide-bound cysteines (C_28_C_35_C_41_C_48_C_54_C_63_) and a highly conserved cluster of negatively charged residues, LRR domain, hinge region (359–402 residues), and 7-transmembrane domain. **(B)** The Maximum Likelihood evolutionary tree of RXFP2 constructed by MEGA X software of representative mammals. The hamster RXFP2 branch was further clustered with the rodentia group. Sequence identities are indicated at right. **(C)** PyMOL 3D-structural analyses localized the signal peptide in the extracellular N-terminus, LRR domain, hinge region, 7-transmembrane domain in the intracellular C-terminus. 3D-conformational structure showed seven alpha-helices interconnected by extracellular and intracellular loops.

Using Clustal Omega multiple sequence alignment and NCBI Protein–BLAST analysis, the amino acid sequences of hamster INSL3 (Fig 3) and RXFP2 (Fig 4) were aligned and characterized structurally from other mammalian species. Consequently, the hamster INSL3 sequence revealed five distinct structural domains (Fig 3A), namely signal peptide (residues 1–15), cleavage site (residues 16 and 17), B-chain (residues 18–51), C-chain (residues 54–98), A-chain (residues 100–125), and a disulfide bond region (residues 109–114). The MEGA X program and multiple sequence alignment were used for a maximum likelihood phylogenetic analysis/JTT matrix-based model of the hamster INSL3 amino acid sequence, confirming a 57–81% identity and homology (Fig 3B) to the amino acid sequences of *B. taurus*, *Capra hircus*, *Sus scrofa*, *Canis lupus familiaris*, *H. sapiens*, *Macaca mulatta*, *M. musculus*, and *R. norvegicus*. The hamster INSL3 demonstrated a high amino acid sequence similarity (80–81%) with rodent species. Robetta and PyMOL 3.0 were used to predict the 3D structural model of hamster INSL3 (Fig 3C), illustrating its classification within the insulin-like hormone superfamily. Spatial alignment results revealed that the 3D structure of each functional hamster INSL3 domain was similar to human INSL3, confirming structural conservation.

Comparative sequence studies and the identification of conserved domains have been conducted on the hamster RXFP2 protein (Fig 4), revealing insights into its structure and evolutionary relationships. The research uncovered six evolutionarily conserved domains (Fig 4A) which can be classified as a signal peptide (1–19 residues), a cleavage site (19–20 residues), and a low-density lipoprotein receptor class A domain (LDLa, 27–64 residues). This LDLa contains six disulfide-bound cysteines (C_28_C_35_C_41_C_48_C_54_C_63_) and a highly conserved cluster of negatively charged residues. Other structural features include a leucine-rich repeat (LRR) domain (LRR1 through LRR10, with residues ranging from 121–358), a hinge region (359–402 residues), and a 7-transmembrane domain (seven TMs with residues ranging from 403–675). Phylogenetic analysis strongly supports a close relationship between hamsters and other mammalian species as well as a sister relationship with rodents. Molecular diversity analysis of the RXFP2 protein showed high degree of similarity with *R. norvegicus* (91.99%) and *M. musculus* (92.81%), with a notably lower homology to *H. sapiens* RXFP2 protein at 82.72% (Fig 4B). The 3D structural model of the hamster RXFP2 indicates its classification within the G protein-coupled, 7-transmembrane receptor (GPCR) family (Fig 4C). Proteins within the same branch display structural domains with high homology, conserved motifs, and similar 3D structures, indicating the robust conservation of RXFP2 proteins.

### Gene expression profiles of the INSL3/RXFP2 signaling pathway

**Fig 5:**
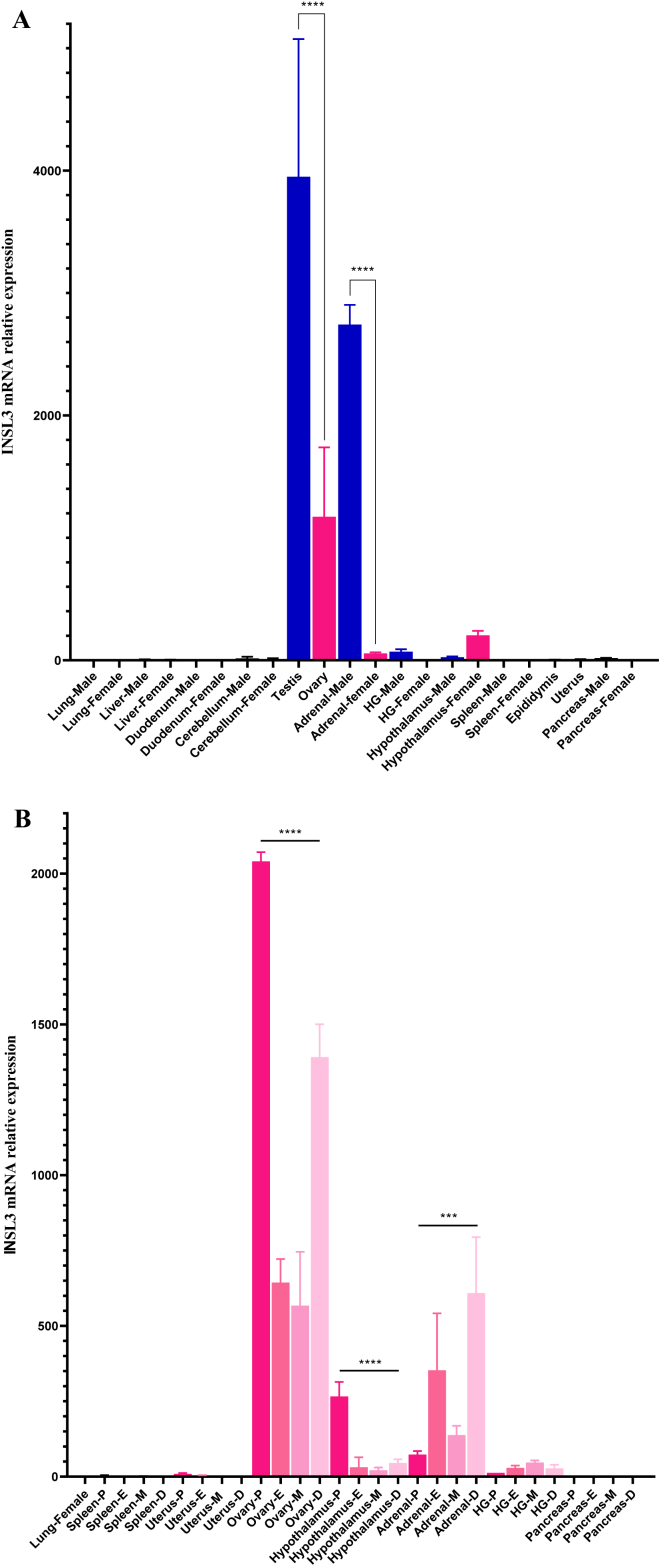
Tissue-specific analysis of INSL3 mRNA via qPCR assays in adult hamster and effects of sex-steroid hormones during estrous cycle. **(A)** A panel of tissues was collected and examined in this study, including the lung, liver, duodenum, cerebellum, testis, ovary, adrenals, HG, hypothalamus, spleen, epididymis, uterus, and pancreas. **(B)** Relative expression profiles were determined during different stages of the estrous cycle such as proestrus P, estrus E, metestrus M, and diestrus **D.** The *INSL3* gene under study was normalized using *ACTB* as the housekeeping gene. The means with the asterisks are statistically significantly different between males (in blue) and females (in pink). The statistical significance was calculated using a one-way analysis of variance (ANOVA), followed by Sidak´s and Tukey’s test (*p* < 0.05). Data are shown as means ± SD (n = 5). **** indicate *p* < 0.0001, *** indicate *p* < 0.001.

**Fig 6:**
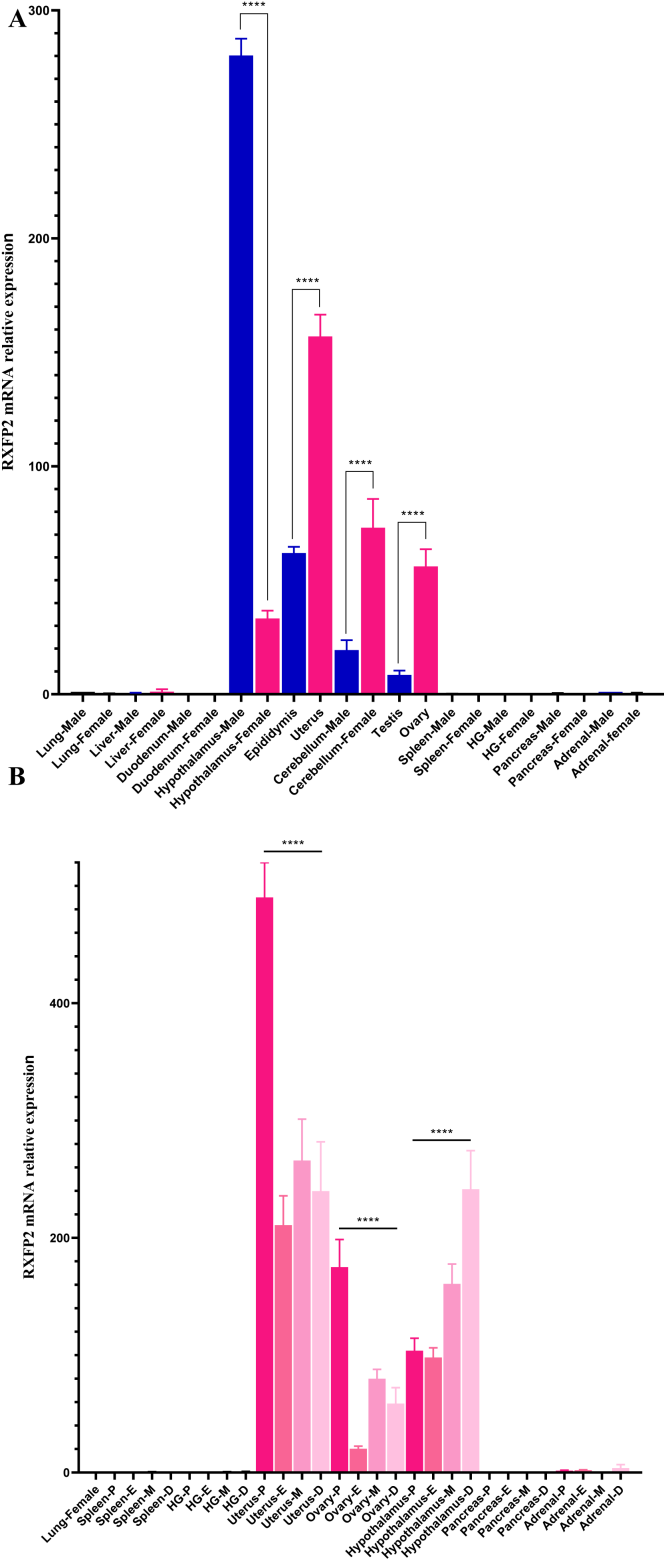
Relative expression levels of RXFP2 mRNA in different tissues and evaluation of sex-steroid hormones during the estrous cycle from adult hamster. **(A)** In males (in blue) and females (in pink) hamsters, the relative gene expression was analyzed in lung, liver, duodenum, hypothalamus, epididymis, uterus, cerebellum, testis, ovary, spleen, HG, pancreas, and adrenals. **(B)** Additionally, differential gene expression analyses were performed at estrous cycle; proestrus P, estrus E, metestrus M, and diestrus **D.** Values are expressed as mean ± SD of five independent qPCR assays (n = 5). *ACTB* gene was used as an internal reference to examine the mRNA levels of RXFP2. Asterisks (****, indicate *p* < 0.0001) above each vertical bar indicate statistical differences, as determined by ANOVA followed by Sidak´s and Tukey’s tests (*p* < 0.05).

To understand the endocrine landscape of the INSL3/RXFP2 signaling pathway, we examined the mRNA expression profiles in adult hamster tissues from both sexes, along with the gene regulation in the estrous cycle, from *INSL3* (Fig 5) and *RXFP2* (Fig 6) genes. Our findings showed significant expression of the INSL3 transcript in the testes, male adrenal glands, and ovaries, whereas lower expression was observed in the female hypothalamus, male HG, female adrenal glands, and male hypothalamus (statistical significance = *p* < 0.0001). Furthermore, no detectable mRNA expressions of INSL3 were observed in tissues such as the lung, liver, duodenum, cerebellum, spleen, pancreas, epididymis, and uterus (Fig 5A). Similarly, differential INSL3 expression was observed in the ovary, adrenal glands, hypothalamus, and HG during the estrous cycle, with the analysis revealing that the INSL3 mRNA expression level was relatively higher in the ovary tissue during the proestrus phase (*p* > 0.0001 and *p* < 0.001). INSL3 expression was not detected in the lung, spleen, uterus, and pancreas across different phases of the estrous cycle (Fig 5B).

To investigate the variations in RXFP2 relative expression profiles in hamster tissues, we performed qPCR reactions using the standard method (Fig 6). The highest relative expression levels were found in the male hypothalamus, uterus, female cerebellum, epididymis, and ovary. In contrast, the least abundant transcripts were detected in the female hypothalamus, male cerebellum, and testis (*p* < 0.0001). Tissue distribution analysis in hamsters showed that RXFP2 mRNA was not detected in the lung, liver, duodenum, spleen, HG, pancreas, and adrenals (Fig 6A). Moreover, differential RXFP2 expression was noticed in the uterus, hypothalamus, and ovary during the estrous cycle. This analysis indicated that RXFP2 mRNA expression levels were significantly higher in uterine tissue during the proestrus phase. Specifically, this uterus had twofold and threefold more RXFP2 mRNA than the female hypothalamus and ovary hamster (*p* < 0.0001), respectively. The adrenal had the lowest mRNA levels during the estrous cycle. Meanwhile, transcripts in the lung, spleen, HG, and pancreas during the estrous cycle were not detected (Fig 6B).

## Discussion

A key goal of comparative and reproductive endocrinology research is the description of hormone/receptor systems, as well as understanding the transcriptional, structural, and evolutionary relationships between hormone and receptor families. Generating knowledge across different endocrine systems or biological models can aid in identifying underlying mechanisms in rare genetic diseases, disorders of sex development, or hormonal strategies for reproduction. In mammalian endocrinology, INSL3/RXFP2 signaling plays a significant role in male reproductive development and fertility; specifically, these two molecules’ functional mechanisms are involved in the testicular descent into the scrotum. In humans, INSL3 and RXFP2 transcripts are primarily expressed in the testicular Leydig cells and the gubernaculum around weeks 10–12 of pregnancy. Mutations in the *INSL3*/*RXFP2* genes are associated with cryptorchidism or undescended testis, a reproductive birth defect characterized by impaired fertility due to spermatogenic maturation arrest [10,23,24]. However, in female mammals, INSL3/RXFP2 signaling has been less explored. Therefore, this study aimed to elucidate and explore the structural and transcriptional landscape of INSL3/RXFP2 signaling in hamster tissues, particularly those relevant to female reproductive endocrinology.

In this study, we employed a molecular cloning assay and a combination of bioinformatics tools, with the objective of understanding the previously unknown INSL3 and RXFP2 protein structures in *Mesocricetus auratus*, known commonly as the Syrian hamster. We identified evolutionarily conserved regions or domains in the hamster’s INSL3 and RXFP2 peptides, indicating these proteins possess a highly conserved phylogenetic relationship across mammals and perform a similarly conserved function [25–29]. Essential to these functions are the signal peptides and the binding domains between INSL3 (B-chain or B-peptide region) and RXFP2 (LRR elements). These LRR elements play a crucial role in the primary recognition of the INSL3 hormone via its B-peptide region [2,30,31]. Similarly, 3D molecular modeling of the hamster’s RXFP2 protein suggests that the conserved LRR sequence is sufficient to form the characteristic arc or horseshoe curvature in proteins containing 20–30 residue repeats [32]. We also identified multiple structural and functional region candidates such as the B-chain, C-chain, A-chain, and disulfide bond region in the INSL3 protein, and relaxin type domains such as LLR regions and 7-transmembrane domain in the RXFP2 protein. This supports the hypothesis that these protein domains maintain a high degree of molecular evolutionary conservation. We have expanded our previous understanding of hamster INSL3/RXFP2 proteins with protein sequence homologies, particularly in higher vertebrate proteins. Interestingly, all these peptides belong to the insulin-like hormone superfamily and receptors for relaxin family peptides, reinforcing the idea that the INSL3/RXFP2 signaling has evolved for endocrine and reproductive processes [33,34].

Previous studies have provided ample evidence that the insulin-like hormone superfamily and receptors for the relaxin family (a G protein-coupled receptors) are vital proteins that regulate multiple reproductive processes including the development of the urogenital tract, testicular function, fertility, and female reproductive physiology [35]. Particularly, the INSL3/RXFP2 signaling system has been shown to act as a key regulator in gubernaculum development, testicular descent during gestation, lactation, pregnancy, and birth in different mammalian species [14,17,27,36,37]. For instance, INSL3/RXFP2 signaling functions as a survival/anti-apoptotic factor in maintaining sperm production in Duroc boar testes [38] and is critical for testicular descent in mice and humans [4,39].

Gene expression analysis of INSL3/RXFP2 in theca interna cell (TIC) and granulosa cell compartments of developing bovine antral follicles and corpora lutea (CL) revealed the importance of both peptides as intrafollicular modulators of TIC function/steroidogenesis. However, expression levels of both INSL3 and RXFP2 proteins in CL were much lower than in TIC, suggesting that functional INSL3/RXFP2 signaling is only weakly operational in CL tissue [18]. Previous studies have also reported INSL3 as a key theca cell-derived growth factor for preantral follicles and that its action is mediated by GDF9 in rats [40]. INSL3 has also been detected in TIC of antral follicles in female mammals of bovines [41], rodents [42], humans [43], and monkeys [44].

In this study, we observed that male INSL3 is specifically expressed in the testis, adrenal, HG, and hypothalamus in adult hamsters. Meanwhile, expression analysis revealed a transcript in the ovary, hypothalamus, and adrenals in female hamsters. These tissues displayed varying mRNA relative expression levels during the estrous cycle. In the ovary, the INSL3 mRNA was overexpressed during proestrus, suggesting that sex-steroid hormones may regulate/induce *INSL3* gene expression in the ovary, hypothalamus, and adrenals. However, low levels of INSL3 mRNA were identified in the hypothalamus and GH in male hamsters compared to the high levels in their testes and adrenals. The results could suggest that local biosynthesis of INSL3 is occurring, which would imply intracrine regulation of cellular functions via INSL3/RXFP2 signaling in these tissues.

Elevated expression of a specific *RXFP2* gene was identified in the hypothalamus, while decreased expression occurred in the epididymis, cerebellum, and testis in relation to the hypothalamus of male hamsters. Interestingly, we found that RXFP2 in female hamsters was expressed in the uterus, cerebellum, ovary, and hypothalamus. Furthermore, the *RXFP2* gene expression in hamsters exhibits significant changes during the estrous cycle. In the literature, limited data have been reported on the impact of RXFP2 on female reproductive physiology and endocrinology. The available data suggest that both RXFP1 and RXFP2 are upregulated in response to reduced uteroplacental blood flow in rats [45]. A later study examined RXFP1 receptor expression in the myometrium of pregnant rats and observed a significant decrease towards the end of gestation, which was influenced by local factors that are derived from the conceptus [46]. This finding complements the results of the original article, which reported upregulation of RXFP1 and RXFP2 in response to uteroplacental restriction. The authors conclude that this may represent a novel mechanism for the activation of spontaneous uterine contractions during labor in this species.

We speculate that male INSL3/RXFP2 signaling in the hypothalamus of adult hamsters is sensitive to the levels of local hypothalamic INSL3 and might regulate fertility via a local feedback loop to control the release of gonadotropin-releasing hormone (GnRH) to secrete luteinizing hormone (LH; responsible for the biosynthesis of testosterone) and follicle-stimulating hormone (FSH; responsible for the spermatogenesis). In the hypothalamus, the gene expression of *INSL3* and *RXFP2* suggests paracrine/autocrine functions of GnRH/LH/FSH signaling during steroidogenesis or spermatogenesis. Furthermore, these results suggest the possible involvement of hypothalamic INSL3 and exclude the involvement of testicular INSL3.

Moreover, in the epididymis, INSL3/RXFP2 signaling might play a crucial role in the maturation, storage, and transport of sperm. We also hypothesize that female INSL3/RXFP2 signaling in the uterus of adult hamsters plays a vital role in the estrous cycle. Importantly, hamster INSL3/RXFP2 signaling might also influence male and female reproductive health in an endocrine (regulation of the hypothalamic-testis axis) and paracrine (regulation of the ovary-uterus axis) manner via sex-steroid hormone regulation, respectively. In this study, the results obtained using the specific gene expression of *INSL3*/*RXFP2* signaling in hamsters contained many strengths and weaknesses that may be addressed in subsequent immunohistochemical assays, RNAscope techniques, or in situ mRNA hybridization techniques. For example, it would have been very interesting to determine what cell types show *INSL3*/*RXFP2* gene expression in female hamsters (e.g., which ovarian region) and male hamsters (e.g., which epididymal regions). Possible examples include the testes and particularly the hypothalamus, adrenal glands (e.g., which hypothalamic nuclei or adrenal zones), and uterus.

The relationship between these reproductive signaling axes and the regulation of sex-steroid hormones in hamsters, including the potential for sex-dimorphic responses, warrants further study – this also extends to humans. Exploring how this signaling likely influences the development of conditions such as endometriosis or uterine abnormalities in humans could provide an interesting avenue for future clinical research. Lastly, additional studies are necessary to elucidate the biological function of the INSL3/RXFP2 signaling in the cerebellum of both female and male hamsters.

## Conclusions

In summary, the present study enhances our understanding of the endocrine INSL3/RXFP2 signaling and the sites of gene expression involved during the reproductive stages of both male and female hamsters. Nevertheless, the potential gene regulation by sex-steroid hormones in male and female hamsters remains to be molecularly characterized, necessitating further research to fully grasp their role in female reproductive physiology, especially in the uterus. Although our findings support an evolutionarily conserved role for INSL3/RXFP2 structure and expression in reproductive endocrinology, it is yet to be established to what extent this signaling function has been altered in the human uterus and cerebellum, as well as its likely association with uterine clinical disorders.
